# First Mitogenome of the Critically Endangered Arabian Leopard (*Panthera pardus nimr*)

**DOI:** 10.3390/ani15111562

**Published:** 2025-05-27

**Authors:** Fahad H. Alqahtani, Ion I. Măndoiu, Badr M. Al-Shomrani, Sulaiman Al-Hashmi, Fatemeh Jamshidi-Adegani, Juhaina Al-Kindi, Andrzej Golachowski, Barbara Golachowska, Abdulaziz K. Al-Jabri, Manee M. Manee

**Affiliations:** 1 National Center for Bioinformatics, King Abdulaziz City for Science and Technology, Riyadh 11442, Saudi Arabia; fqahtani@kacst.gov.sa (F.H.A.); shomrani@kacst.gov.sa (B.M.A.-S.); 2 Advanced Agricultural and Food Technologies Institute, King Abdulaziz City for Science and Technology, Riyadh 11442, Saudi Arabia; 3 Computer Science and Engineering Department, University of Connecticut, Storrs, CT 06269, USA; 4 Laboratory for Stem Cell and Regenerative Medicine, Natural and Medical Sciences Research Center, University of Nizwa, Nizwa 616, Oman; 5 Royal Court Affairs, Muscat 113, Oman

**Keywords:** *Panthera pardus nimr*, Arabian leopard, mitochondrial genome, phylogeny, endangered species

## Abstract

The Arabian leopard (*Panthera pardus nimr*), a critically endangered subspecies endemic to the Arabian Peninsula, faces severe threats from habitat loss, prey depletion, and inbreeding, with fewer than 200 individuals remaining. Genomic resources for this subspecies have been scarce, limiting insights into its evolutionary history and conservation needs. Here, we present the first complete mitochondrial DNA (mtDNA) sequence of *P. pardus nimr*, derived from a wild-born male sampled at the Oman Wildlife Breeding Centre in 2023. Using PacBio HiFi sequencing, we assembled a 16,781 bp mitogenome (GenBank: PQ283265) comprising 13 protein-coding genes, 22 tRNA genes, two rRNA genes, and a control region, with a GC content of 40.94%. Phylogenetic analysis, incorporating 17 *Panthera* mtDNA sequences, positions *P. pardus nimr* closest to African leopard populations from South Africa (*Panthera pardus*), while distinguishing it from Asian subspecies (*P. pardus japonensis* and *P. pardus orientalis*). This mitogenome reveals conserved vertebrate mitochondrial structure and provides a critical tool for studying *Panthera* genus evolution. Moreover, it enhances conservation genetics efforts for *P. pardus nimr* by enabling population structure analysis and informing breeding strategies to strengthen its survival.

## Simple Summary

The Arabian leopard (*Panthera pardus nimr*), a critically endangered subspecies endemic to the Arabian Peninsula, faces threats from habitat loss and population decline, with fewer than 200 individuals remaining in the wild. Genomic resources for this species have been scarce, limiting conservation efforts. This study presents the first complete mitochondrial genome sequence of the Arabian leopard, derived from a wild-born male sampled in Oman in 2023 using PacBio HiFi sequencing. The 16,781 bp mitogenome comprises 13 protein-coding genes, 22 tRNA genes, two rRNA genes, and a control region. Phylogenetic analysis reveals a close relationship with African leopard populations, potentially indicating historical connectivity between African and Arabian lineages. This genomic resource offers critical insights into the leopard’s evolutionary history and genetic diversity, enhancing conservation strategies such as genetic monitoring, captive breeding, and habitat restoration to ensure the species’ long-term survival.

## 1. Introduction

The Arabian leopard (*Panthera pardus nimr*), known locally as Al nimr al-arabi, is the smallest of the eight recognized subspecies of *Panthera pardus*, with adults typically weighing between 18 and 34 kg [1]. Endemic to the Arabian Peninsula, this elusive felid is classified as critically endangered on the IUCN Red List of Threatened Species, with an estimated wild population of fewer than 200 individuals [2,3]. Its historical range once extended across the rugged arid and semi-arid terrains of the Red Sea coast, spanning approximately 1700 km from Haqel in northwest Saudi Arabia through mountainous regions of Yemen, Hadramout, northeast Oman, and into the eastern mountains of the United Arab Emirates [1,4,5]. Today, however, this range has contracted significantly due to a suite of anthropogenic pressures, leaving fragmented populations in isolated desert habitats [3,6,7].

The Arabian leopard faces multifaceted threats that imperil its survival. Overgrazing by domestic livestock, urban expansion into remote mountainous areas, and infrastructure development in the southwestern highlands of Saudi Arabia have drastically reduced suitable habitat [8,9]. Additional stressors include mining activities, gravel extraction, a declining prey base, competition with humans and livestock, retributive killings following human-wildlife conflict, as well as exposure to enteropathogens and diseases, including prion disorders, affecting *Panthera pardus* subspecies [9,10,11,12,13,14,15]. In particular, Buddhirongawatr et al. [13], Stewart et al. [14], Rampacci et al. [15] have emphasized that small and fragmented leopard populations face compounding risks from health-related pressures. Although the disease-related data cited do not specifically examine Arabian leopards, evidence from captive leopards, including *P. pardus* individuals housed in zoological institutions, suggests that such threats may be broadly relevant across the species. These health stressors, in conjunction with anthropogenic pressures, may contribute to continued population decline and further compromise long-term viability. Despite these concerns, the genetic basis of disease susceptibility and the extent of mitochondrial diversity in Arabian leopards remain poorly characterized. A complete mitogenome reference provides foundational insight into maternal lineage history and supports broader conservation genomics, particularly when integrated with health and ecological surveillance. These pressures have not only diminished population sizes but also disrupted connectivity between subpopulations, resulting in reduced genetic diversity and increased inbreeding [12]. Such genetic erosion, a common plight among endangered species, compromises adaptive capacity and reproductive success, heightening extinction risk [10,12].

Mitochondrial DNA (mtDNA) sequencing has emerged as a powerful tool for understanding population dynamics and evolutionary relationships in endangered species [16]. The mitochondrial genome, with its high mutation rate and maternal inheritance, offers insights into historical population structure, gene flow, and phylogenetic divergence [17]. For *Panthera* species, genomic and mitogenomic analyses have elucidated evolutionary histories, including the divergence of the lion (*Panthera leo*) from its close relative, the leopard (*Panthera pardus*), approximately 1.95 million years ago [18]. Within *Panthera pardus*, subspecies like the Amur leopard (*P. pardus orientalis*) and Sri Lankan leopard (*P. pardus kotiya*) have benefited from mtDNA analyses that inform conservation strategies [19,20]. However, prior to this study, no complete mitochondrial genome sequence existed for *P. pardus nimr*, representing a critical gap in genomic resources for this imperiled subspecies.

The absence of mitogenomic data has hindered efforts to fully understand the Arabian leopard’s phylogenetic position within the *Panthera* genus and assess its genetic health, information that is vital for effective conservation planning. Previously, mapping of raw whole-genome sequenced data from two captive Arabian leopards onto a domestic cat reference genome revealed the subspecies’ phylogenetic position as sister to other Asian leopard subspecies, a prolonged population decline causing low genetic diversity, high inbreeding, and introgression from African leopards (*P. pardus pardus*), crucial insights that earlier studies using mitochondrial DNA fragments and limited nuclear loci lacked the resolution to fully resolve [12]. Further establishing a comprehensive mitogenome can provide a baseline for assessing population structure, identifying unique genetic signatures, and detecting deleterious mutations, all of which are essential for designing captive breeding and reintroduction programs [21]. Moreover, comparative analyses with other *Panthera* mitogenomes could clarify the evolutionary relationships of *P. pardus nimr*, potentially revealing historical connectivity with African or Asian leopard lineages.

This study addresses these gaps by sequencing, assembling, and annotating the complete mitochondrial genome of *Panthera pardus nimr* using a blood sample obtained from a wild-born individual in Oman. High-fidelity long-read sequencing (PacBio HiFi) enabled accurate reconstruction of the mitogenome and analysis of its gene content, structure, and base composition. Comparative phylogenetic analysis was then used to assess the evolutionary placement of this critically endangered subspecies relative to other *Panthera* taxa. The resulting mitogenome provides a foundational genomic resource for evolutionary research and conservation planning.

## 2. Materials and Methods

### 2.1. Sample Collection and Ethical Approval

**Figure 1:**
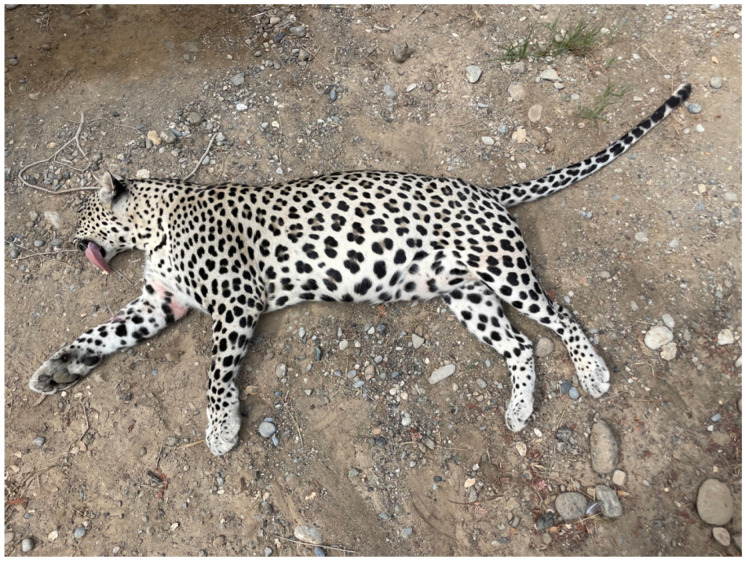
Reference photograph of the Arabian leopard (*Panthera pardus nimr*), taken by the author Andrzej Golachowski at the Directorate General of Veterinary Services, Diwan of the Royal Court (latitude: 23.66° N, longitude: 58.19° E).

On 4 September 2023, a male Arabian leopard (*Panthera pardus nimr*), born in 2011, underwent a health check at the Oman Wildlife Breeding Centre in Barka, Oman before being relocated to the Directorate General of Veterinary Services, Diwan of the Royal Court (latitude: 23.66° N, longitude: 58.19° E). The leopard was chemically immobilized using a mixture of tiletamine and zolazepam (Telazol, Zoetis Inc., Parsippany-Troy Hills, NJ, USA) and medetomidine (Domitor, Orion Pharma, Espoo, Finland), prepared by combining 5 mL of Domitor with one vial of Telazol to achieve a concentration of 50 mg zolazepam, 50 mg tiletamine, and 1 mg medetomidine per mL (Figure 1). This solution was administered via 1.5 mL dart syringes with a 1.50 × 30 mm collared needle fired from a CO_2_-powered rifle. Following anesthesia, blood was collected from the left jugular vein into EDTA and serum tubes. After 20 min, the anesthesia was reversed by administering 1 mL (5 mg) of atipamezole (Antisedan, Orion Pharma, Espoo, Finland) and 8 mL (0.80 mg) of flumazenil (Flumazenil, B. Braun Melsungen AG, Melsungen, Germany) via intramuscular injection, and the leopard was returned to its enclosure. This wild-born individual was selected for sampling to represent natural genetic variation, and the health check was part of routine veterinary care prior to relocation. The immobilization process ensured minimal stress and safe handling, with drug dosages calculated based on the animal’s estimated body weight. Ethical approval was granted under ethics number VCGSR/AREC/05/2023, ensuring compliance with international standards for animal research.

### 2.2. DNA Extraction

Genomic DNA was extracted from the collected blood sample using the PureLink™ Genomic DNA Mini Kit (Thermo Fisher Scientific, Waltham, MA, USA) according to the manufacturer’s protocol. Briefly, 20 μL of Proteinase K was added to a microcentrifuge tube, followed by 200 μL of whole blood and 20 μL of RNase A. The mixture was gently vortexed and incubated at room temperature for 2 min. Subsequently, 200 μL of lysis/binding buffer was added, followed by incubation at 55 °C for 10 min to facilitate cell lysis and protein digestion. After lysis, 200 μL of 100% ethanol was added, and the lysate was transferred to a spin column. The column was centrifuged and washed sequentially with Wash Buffers 1 and 2, then eluted in 70 μL of elution buffer by centrifugation at 14,000× *g* for one minute. The extraction process was optimized to maximize yield and quality from a limited blood volume. Proteinase K and RNase A were used to ensure efficient protein and RNA degradation, and purification was enhanced through carefully controlled centrifugation conditions. DNA concentration and purity were assessed by UV absorbance at 260 nm and 280 nm using a spectrophotometer, and integrity was confirmed by the presence of high-molecular-weight bands on a 1% agarose gel via electrophoresis. To preserve DNA integrity, the sample was stored at −20 °C. The extracted DNA was deposited at the Laboratory for Stem Cells and Regenerative Medicine, Natural and Medical Sciences Research Center, University of Nizwa, Oman (contact person: Sulaiman Al-Hashmi, sahashmi@unizwa.edu.om) under voucher number LSCRM-L-02-04 (latitude: 22.91° N, longitude: 57.67° E).

### 2.3. Library Preparation and Sequencing

DNA samples were prepared for sequencing using the PacBio SMRTbell Template Preparation Kit (v3.0) according to the manufacturer’s protocol. Sequencing was performed on the PacBio Revio system, selected for its ability to generate long, high-fidelity reads and hence to provide robust coverage for assembling a complete mitochondrial genome. Sequencing was outsourced to Macrogen, South Korea. The generated HiFi reads had an average quality score of Q29. Two samples were processed, producing a combined total of 162.90 Gbp of high-quality data, which were deposited in the NCBI Sequence Read Archive (SRA accession: PRJNA1091853).

### 2.4. Mitochondrial Genome Assembly and Annotation

MitoHiFi v3.2.2 [22] was used for assembly of the mitochondrial genome, and the resulting sequence was annotated with the MITOS2 web server [23]. This annotation was visualized using OGDRAW [24]. The assembly process utilized MitoHiFi’s reference-guided algorithm to extract mitochondrial sequences from the whole-genome HiFi reads, filtering out nuclear mitochondrial pseudogenes based on long-read accuracy and coverage depth. Ultimately, a single contiguous mitochondrial genome sequence was assembled for *P. pardus nimr*.

### 2.5. Phylogenetic Analysis

A total of 17 *Panthera* mitochondrial genome sequences, including an outgroup species (*P. leo leo*), were retrieved from GenBank and used to position the mitogenome of *P. pardus nimr* in a phylogenetic context. The retrieved sequences included three from *P. pardus japonensis*, ten from *P. pardus*, two from *P. pardus orientalis*, two from *P. pardus kotiya*, and one from *P. leo leo*, providing a comprehensive dataset for comparison. The phylogenetic tree was constructed using the basic local alignment search tool (BLAST; accessed on 5 January 2025 from https://blast.ncbi.nlm.nih.gov/Blast.cgi) with the fast minimum evolution method and a maximum sequence difference of 0.75, and was visualized using the iTOL v6 online tool [25]. To assess genetic variability and evolutionary relationships within Panthera pardus, we used the BLAST Tree View tool, which applies the Jukes–Cantor model to compute a nucleotide distance matrix and constructs a phylogenetic tree using the fast minimum evolution (FastME) algorithm [26]. The iTOL visualization tool displayed branch lengths and taxonomic relationships rooted on the outgroup. It should be noted that evident discrepancies exist in the nomenclature of subspecies across published articles. To maintain scientific integrity and consistency with the primary data, we have elected to adopt the subspecies names as originally submitted by the authors to the NCBI GenBank database, and, where applicable, as presented in their related publications.

## 3. Results

### 3.1. Mitogenome Structure and Composition

**Figure 2:**
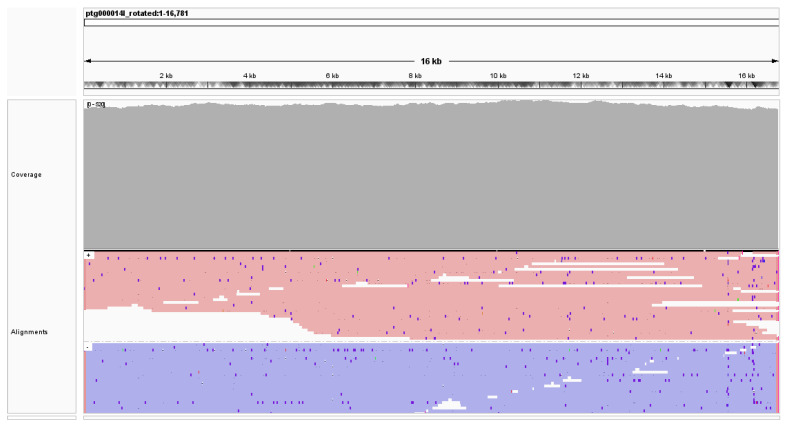
Summary of sequencing coverage and alignment data for the mitochondrial genome of *Panthera pardus nimr*. Average coverage computed using samtools [29] was approximately 503×. The coverage and alignments tracks visualized in IGV [27] show minimal coverage variation, with reads uniformly aligned across the assembled mitochondrial genome.

The mitochondrial genome assembly of *Panthera pardus nimr* is of high quality in sequence and alignment, as evidenced by both quantitative metrics and read alignment patterns. Figure 2 shows the IGV [27] visualization of alignments generated using minimap2 [28] for all PacBio reads that have 90% or more of their bases aligned to the mitogenome assembled by MitoHiFi [24]; these high-quality alignments exclude potential contamination with alignments of reads generated from nuclear mitochondrial (NUMT) DNA segments. The average coverage by high-quality alignments, as computed using samtools [29], was approximately 503×. As shown by the “Coverage” track in Figure 2, there is minimal variation in coverage across the entire 16,781 bp. Additionally, the coverage plot shows no significant coverage gaps or drops below 500×, confirming the reliability of the sequencing data used for mitochondrial genome reconstruction. Alignment analysis further supports consistent and strand-balanced read alignment across the entire mitogenome, with red lines indicating forward-strand alignments and purple lines representing reverse-strand alignments in the “Alignments” track of Figure 2. The uniformity and strand balance of read alignments underscore the quality of the assembled mitogenome, which facilitates accurate downstream analyses of genetic structure and evolutionary relationships.

**Figure 3:**
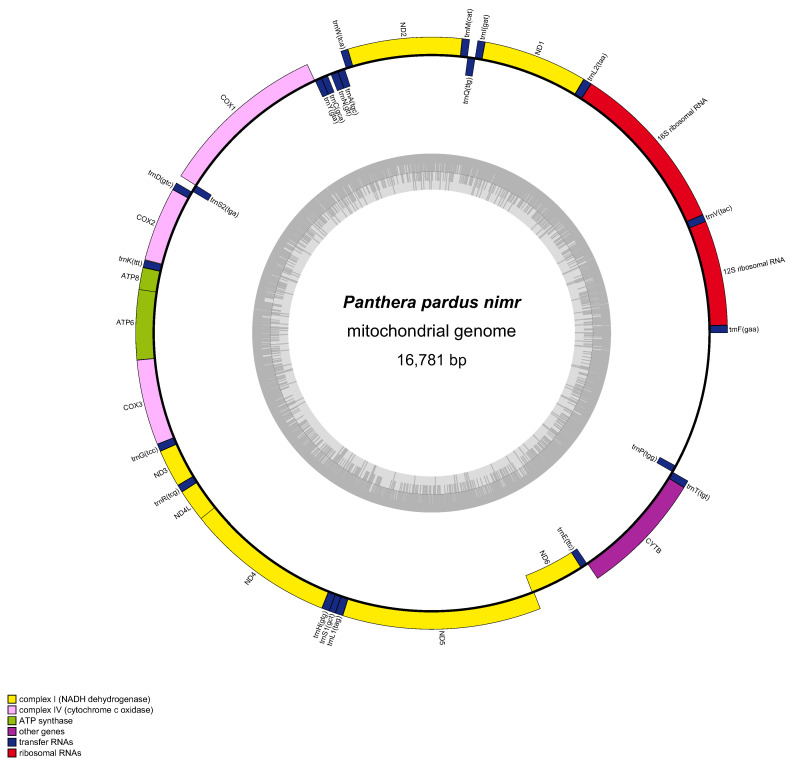
Organization of the complete mitogenome of *Panthera pardus nimr* (Arabian leopard). Colour blocks represent distinct genes. Blocks outside the circle indicate genes on the heavy strand, and blocks inside indicate genes on the light strand. The inner grey-coloured circle represents the GC content graph.

The mitochondrial genome of the Arabian leopard comprises a complete sequence of 16,781 base pairs (bp), encoding a total of 37 genes. This includes 13 protein-coding genes (PCGs), 22 transfer RNA (tRNA) genes, and two ribosomal RNA (rRNA) genes identified as 12S rRNA and 16S rRNA alongside an origin of light-strand replication (OL) and a single control region critical for replication and transcription (Table 1). The nucleotide composition comprises A: 31.59%, T: 27.47%, G: 14.09%, and C: 26.85%, resulting in a total GC content of 40.94%. With regard to gene distribution across strands, the nad6 subunit gene and six tRNA genes (encoding tRNA-GLU, tRNA-ALA, tRNA-ASN, tRNA-CYS, tRNA-TYR, and tRNA-SER) are situated on the light (L) strand, while the remaining 30 genes, comprising all other PCGs, tRNAs, and rRNAs as well as the control region reside on the heavy (H) strand. Among the PCGs, the longest is nad5 at 1821 bp, which encodes a 606-amino acid protein, whereas the shortest is atp8 at 204 bp, which encodes a 67-amino acid protein. Start codon analysis revealed that ten PCGs (cox1, cox2, cox3, atp6, atp8, nad1, nad4, nad4l, nad6, and cytb) initiate with ATG, nad2 with ATC, and nad3 and nad5 with ATA. Stop codon usage indicated that eight PCGs (cox1, cox2, atp8, tp6, nad1, nad4l, nad5, and nad6) terminate with TAA, while three (nad3, cox3, and nad4) exhibit incomplete stop codons (T), likely completed to TAA via post-transcriptional polyadenylation. The remaining PCGs utilize alternative stop codons: AGA for cytb and TAG for nad2. The OL, positioned between the genes encoding tRNA-ASN and tRNA-CYS, spans 37 bp and serves as the initiation site for L-strand replication. Figure 3 presents a detailed circular map illustrating the gene arrangement and strand orientation, providing a comprehensive visual representation of this genomic architecture.

### 3.2. Phylogenetic Relationships

**Figure 4:**
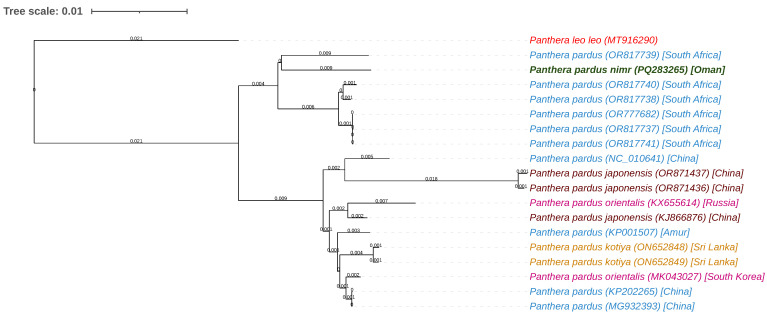
Phylogenetic tree of *Panthera pardus nimr* (Arabian leopard) and related subspecies based on mitochondrial genome sequences. Branch lengths are indicated next to the branches. The Arabian leopard (accession PQ283265) is highlighted in green, the outgroup species *Panthera leo leo* in red, *Panthera pardus* in blue, and other *P. pardus* subspecies in distinct colors.

A maximum-likelihood phylogenetic tree constructed using the mitochondrial genome of *Panthera pardus nimr* and 18 other *Panthera* mitochondrial sequences retrieved from GenBank elucidated the evolutionary relationships among these taxa (Table 2). The dataset included three sequences of *P. pardus japonensis* (Chinese leopard), ten sequences of *P. pardus* (leopard, primarily from South Africa), two sequences of *P. pardus orientalis* (Amur leopard), two sequences of *P. pardus kotiya* (Sri Lankan leopard), one unspecified *P. pardus* sequence, and the outgroup species *Panthera leo leo* (African lion) (Table 2). Generated via BLAST pairwise alignment with the fast minimum evolution method and a maximum sequence difference threshold of 0.75, the tree revealed distinct clustering patterns, as presented in Figure 4. The *P. pardus nimr* sequence, highlighted in green (PQ283265), exhibited the closest phylogenetic affinity with *P. pardus* sequences from South Africa, including Malalane (OR817739), Skukuza (OR817740), Lydenburg (OR817738), Sabi Sands (OR777682), Middelburg (OR817737), and Piet Retief (OR817741); the short branch lengths indicate pairwise sequence similarities in excess of 99% and hence a tight evolutionary linkage and minimal divergence between the Arabian and African leopard populations, potentially indicative of recent gene flow or historical connectivity. In contrast, *P. pardus nimr* diverged significantly from *P. pardus japonensis* and *P. pardus orientalis*, forming separate Asian clades; these longer branch lengths represent distinct evolutionary trajectories and establish the Arabian leopard as part of a separate lineage within the *Panthera pardus* species complex. Meanwhile, the six sequences collected from locations in China, including those representing the Chinese leopard (*P. pardus japonensis*) (OR871437, OR871436, KJ866876), exhibited considerable intra-subspecies diversity, as evidenced by their varied branch lengths. In contrast, the Amur leopard (*P. pardus orientalis*) sequences (KX655614, MK043027) formed a tighter subgroup with shorter internal branches, indicating lower genetic variation. The names of species and subspecies herein are presented as submitted by the original authors to GenBank, as outlined in the Methods section.

The phylogenetic analysis further delineated the positions of additional *Panthera pardus* lineages and the outgroup, thereby providing a comprehensive evolutionary framework for the genus. Specifically, the *P. pardus kotiya* sequences from Sri Lanka (ON652848, ON652849) and the unspecified *P. pardus* sequence (KP001507) occupied intermediate phylogenetic positions, distinguished by moderate branch lengths relative to the African–Arabian and Asian clusters; this suggests greater divergence from the primary groups and hence a unique evolutionary history or potential historical admixture. The outgroup, *P. leo leo* (MT916290), was positioned at the root of the tree and exhibited the longest branch length, signifying the greatest sequence divergence from all *P. pardus* lineages and confirming its taxonomic separation within *Panthera*. Ultimately, this phylogenetic arrangement offers a robust basis for inferring speciation dynamics within *Panthera pardus*, particularly a closer evolutionary relationship between the Arabian and African leopard populations as compared to Asian leopard subspecies.

## 4. Discussion

The Arabian leopard (*Panthera pardus nimr*) is listed as critically endangered by the International Union for Conservation of Nature (IUCN) [2,36], with its current distribution restricted to fragmented habitats in the Arabian Peninsula, including Oman, Saudi Arabia, and Yemen, as documented in recent conservation assessments [37]. In Saudi Arabia, the species has experienced a drastic decline, with no confirmed sightings since 2014, and extensive surveys suggest it may be functionally extinct in the region due to habitat fragmentation, prey depletion, and human–wildlife conflict [5,38,39,40]. This alarming trend emphasizes the need for advanced conservation tools to support recovery efforts across the Arabian leopard’s range [39,40]. This study presents the first complete mitochondrial genome sequence of *P. pardus nimr*, assembled from a wild-born individual sampled in Oman on 4 September 2023, which represents a seminal genomic resource for this subspecies and marks a significant advancement in its genetic characterization.

In particular, the mitochondrial genome data from this study provide a high-resolution genomic baseline that enables precise analysis of maternal genetic structure, phylogenetic relationships, and population differentiation, addressing critical knowledge gaps essential for mitigating extinction risk amid severe habitat loss and isolation across the Arabian Peninsula [37,41]. This genomic resource offers direct applications for the species’ recovery through facilitating the identification of unique genetic signatures and deleterious mutations vital for designing targeted captive breeding and reintroduction programs, guiding mate selection to maximize genetic diversity through phylogenetic positioning, and informing habitat restoration and population connectivity initiatives to enhance survival prospects under ongoing environmental pressures [19,41]. While mtDNA provides valuable insights into maternal lineage and population structure, it should be complemented with nuclear DNA analysis to fully assess genetic diversity for optimizing breeding programs.

Characterization of the *Panthera pardus nimr* mitogenome revealed it to span a total length of 16,781 base pairs and encode 37 genes, comprising 13 protein-coding genes, 22 transfer RNA genes, and two ribosomal RNA genes (12S and 16S); it also featured an origin of light-strand replication and a control region. This structure closely resembles the mitochondrial genomes of other *Panthera* species in length, including the Amur leopard (*P. pardus orientalis*) at 16,964 bp [35], the South African leopard (*P. pardus*) at approximately 16,678 bp [42], and the snow leopard (*Panthera uncia*) at 16,773 bp [43], as well as in conserved gene order and identical gene counts [44]. Notably, the *P. pardus nimr* mitogenome exhibits a base composition of A: 32.00%, T: 27.50%, C: 26.85%, and G: 14.09%, reflecting an AT bias (59.50%) typical of felid mitochondrial genomes, including those of *P. pardus* (A: 31.80%, T: 27.40%) and *P. uncia* (A: 31.90%, T: 27.10%) [43,44]. Among the protein-coding genes, nad5 is the longest (1821 bp), while atp8 is the shortest (204 bp). All genes were encoded on the heavy strand except for nad6 and eight tRNA genes on the light strand, mirroring the organization in *P. uncia* [43].

Our phylogenetic analysis of mitogenomes positioned *P. pardus nimr* within the *Panthera pardus* species complex and has significant evolutionary and conservation implications. The Arabian leopard’s close mitochondrial affinity with African leopard populations (*P. pardus*), particularly those from South Africa, suggests historical connectivity between these lineages, potentially reflecting past dispersal across the Arabian Peninsula and Africa. However, further nuclear genomic studies remain necessary to confirm gene flow between these populations [19,42]. In contrast, the phylogeny showed distinct divergence of the Arabian leopard from Asian leopard subspecies, highlighting the complex evolutionary history of *P. pardus* and geographic isolation of the Arabian leopard. Additional phylogenetic studies based on mitochondrial DNA also support deep divergence between African and Asian leopard lineages and emphasize the distinct evolutionary paths of regional subspecies, such as the Amur and Javan leopards [45,46,47]. Finally, the clear separation of *Panthera leo leo* as an outgroup reinforces the deep taxonomic divergence within *Panthera*, providing a robust framework for understanding species relationships [42].

In combination, these phylogenetic insights and mitochondrial genome data offer a powerful tool for conservation, enabling the design of breeding programs that maximize genetic diversity and restore population connectivity. Utilizing this information will allow conservationists to address the Arabian leopard’s vulnerability to extinction, enhancing its long-term survival through informed management and habitat restoration initiatives across its native range [41,48].

While this study presents the first complete mitochondrial genome of *P. p. nimr*, it is based on a single individual. Consequently, the sequence may not fully capture the subspecies’ mitochondrial diversity, and individual-specific features may still be present. This highlights the importance of conducting future studies involving multiple individuals to assess intra-population variation and confirm the phylogenetic patterns observed. To explore the potential for expanding our analysis, we examined two publicly available raw whole-genome sequencing datasets for *Panthera pardus nimr*: a male sample (SRR23089148) and a female sample (SRR23089147), both generated using Illumina short-read technology. The mitogenomes of these samples were assembled using two tools, SMART2 [49] and NOVOPlasty v.4.3.3 [50]. However, both tools produced incomplete and fragmented contigs. This outcome is likely due to the presence of nuclear mitochondrial DNA segments (NUMTs) and the inherent limitations of short-read sequencing technologies. Furthermore, the phylogenetic analysis includes only a subset of the currently recognized *P. pardus* subspecies, owing to the limited availability of complete mitochondrial genomes in public databases. Subspecies such as *P. p. delacouri*, *P. p. fusca*, *P. p. saxicolor*, and *P. p. tulliana* are currently unrepresented, which may constrain the resolution of phylogenetic relationships and limit inferences about subspecies divergence. To address these limitations, future research should incorporate both mitochondrial and nuclear genomic data from multiple individuals across all subspecies and utilize long-read sequencing technologies to generate high-quality assemblies that will enable a more comprehensive understanding of *P. pardus* evolutionary history and genetic diversity.

## 5. Conclusions

This study presents the first complete mitochondrial genome of the critically endangered Arabian leopard (*Panthera pardus nimr*), sequenced from a wild-born individual in Oman, revealing a conserved structure of 37 genes. Phylogenetic analysis based on a maximum likelihood tree indicated a close affinity with African leopard populations from South Africa, suggesting that Arabian and African leopards may share a more recent common ancestor and comprise a potential African–Arabian clade distinct from their more divergent Asian counterparts. This genomic milestone not only enriches our understanding of the Arabian leopard’s evolutionary history but also highlights the importance of genetic research in addressing the conservation challenges faced by the subspecies, whose fragmented habitats in Oman, Saudi Arabia, and Yemen are under severe threat from habitat loss and human pressures.

All told, our findings provide a foundational genomic resource that could significantly aid conservation efforts, such as captive breeding and habitat restoration, which may potentially support long-term survival of *P. pardus nimr* across the Arabian Peninsula. The observed divergence from Asian leopard subspecies such as *P. pardus japonensis* and *P. pardus orientalis* emphasizes the unique evolutionary trajectory of the Arabian leopard, which may inform conservation strategies by highlighting the need to preserve its distinct genetic lineage within the *Panthera pardus* species complex. Furthermore, this study sets a precedent for future genomic research on endangered felids, offering a framework for integrating mitochondrial data into broader conservation initiatives, which may prove instrumental in safeguarding the biodiversity of the *Panthera* genus in the face of global decline.

## Figures and Tables

**Table 1:** Annotation of the *Panthera pardus nimr* mitochondrial genome.

Gene	Type	Product	Start (bp)	End (bp)
*trnF(gaa)*	tRNA	tRNA-PHE	1	71
*rrnS*	rRNA	s-rRNA	72	1033
*trnV(tac)*	tRNA	tRNA-VAL	1032	1099
*rrnL*	rRNA	l-rRNA	1100	2674
*trnL2(taa)*	tRNA	tRNA-LEU	2676	2750
*nad1*	CDS	NADH dehydrogenase subunit 1	2753	3709
*trnI(gat)*	tRNA	tRNA-ILE	3709	3777
*trnQ(ttg)*	tRNA	tRNA-GLN	3848	3775
*trnM(cat)*	tRNA	tRNA-MET	3850	3918
*nad2*	CDS	NADH dehydrogenase subunit 2	3919	4962
*trnW(tca)*	tRNA	tRNA-TRP	4961	5029
*trnA(tgc)*	tRNA	tRNA-ALA	5113	5045
*trnN(gtt)*	tRNA	tRNA-ASN	5187	5115
*trnC(gca)*	tRNA	tRNA-CYS	5285	5221
*trnY(gta)*	tRNA	tRNA-TYR	5351	5286
*cox1*	CDS	cytochrome c oxidase subunit 1	5353	6897
*trnS2(tga)*	tRNA	tRNA-SER	6963	6895
*trnD(gtc)*	tRNA	tRNA-ASP	6970	7038
*cox2*	CDS	cytochrome c oxidase subunit 2	7039	7722
*trnK(ttt)*	tRNA	tRNA-LYS	7726	7792
*atp8*	CDS	ATP synthase F0 subunit 8	7794	7997
*atp6*	CDS	ATP synthase F0 subunit 6	7955	8635
*cox3*	CDS	cytochrome c oxidase subunit 3	8635	9419
*trnG(tcc)*	tRNA	tRNA-GLY	9419	9487
*nad3*	CDS	NADH dehydrogenase subunit 3	9488	9834
*trnR(tcg)*	tRNA	tRNA-ARG	9835	9903
*nad4l*	CDS	NADH dehydrogenase subunit 4L	9904	10,200
*nad4*	CDS	NADH dehydrogenase subunit 4	10,194	11,571
*trnH(gtg)*	tRNA	tRNA-HIS	11,572	11,641
*trnS1(gct)*	tRNA	tRNA-SER	11,642	11,700
*trnL1(tag)*	tRNA	tRNA-LEU	11,701	11,770
*nad5*	CDS	NADH dehydrogenase subunit 5	11,771	13,591
*nad6*	CDS	NADH dehydrogenase subunit 6	14,102	13,575
*trnE(ttc)*	tRNA	tRNA-GLU	14,171	14,103
*cytb*	CDS	cytochrome b	14,175	15,314
*trnT(tgt)*	tRNA	tRNA-THR	15,315	15,384
*trnP(tgg)*	tRNA	tRNA-PRO	15,451	15,385

**Table 2:** Mitochondrial genome sequences utilized in phylogenetic analysis of *Panthera pardus nimr* and related taxa.

	Accession Number	Species	Common Name	Collection Location	Reference
1	PQ283265	*Panthera pardus nimr*	Arabian leopard	Oman	This study, 2025
2	MT916290	*Panthera leo leo*	Northern lion	Not specified	Unpublished
3	OR817739	*Panthera pardus*	Leopard	South Africa (Malalane)	Tensen and Camacho [30]
4	OR817740	*Panthera pardus*	Leopard	South Africa (Skukuza)	Tensen and Camacho [30]
5	OR817738	*Panthera pardus*	Leopard	South Africa (Lydenburg)	Tensen and Camacho [30]
6	OR777682	*Panthera pardus*	Leopard	South Africa (Sabi Sands)	Tensen and Camacho [30]
7	OR817737	*Panthera pardus*	Leopard	South Africa (Middelburg)	Tensen and Camacho [30]
8	OR817741	*Panthera pardus*	Leopard	South Africa (Piet Retief)	Tensen and Camacho [30]
9	NC_010641	*Panthera pardus*	Leopard	Not specified	Unpublished
10	KP001507	*Panthera pardus*	Leopard	Not specified	Bertola et al. [31]
11	KP202265	*Panthera pardus*	Leopard	Not specified	Unpublished
12	MG932393	*Panthera pardus*	Leopard	China	Ren et al. [32]
13	OR871437	*Panthera pardus japonensis*	North Chinese leopard	China (Laosicheng)	Zhang et al. [33]
14	OR871436	*Panthera pardus japonensis*	North Chinese leopard	China (Laosicheng)	Zhang et al. [33]
15	KJ866876	*Panthera pardus japonensis*	North Chinese leopard	China (Huangling)	Dou et al. [34]
16	KX655614	*Panthera pardus orientalis*	Amur leopard	Not specified	Unpublished
17	MK043027	*Panthera pardus orientalis*	Amur leopard	South Korea	Kim et al. [35]
18	ON652848	*Panthera pardus kotiya*	Sri Lankan leopard	Sri Lanka	Sooriyabandara et al. [20]
19	ON652849	*Panthera pardus kotiya*	Sri Lankan leopard	Sri Lanka	Sooriyabandara et al. [20]

## Data Availability

The complete mitochondrial genome sequence of *P. pardus nimr* generated in this study is openly available in GenBank under accession number PQ283265. The associated BioProject, SRA, and Bio-Sample numbers are PRJNA1091853, SRX27330393, and SAMN40614674, respectively, ensuring traceability and accessibility of the raw data and assembled sequence.

## References

[1] Spalton J., Willis D. (1999). The status of the Arabian leopard in Oman: First results of the Arabian leopard survey. Nat. Hist. Oman.

[2] Mallon D., Budd K. (2011). Regional Red List Status of Carnivores in the Arabian Peninsula. https://scholar.google.com/scholar_lookup?title=Regional%20Red%20list%20status%20of%20Carnivores%20in%20the%20arabian%20Peninsula&author=D.%20Mallon&publication_year=2011.

[3] Dunford C.E., Martins Q.E., Mann G.K., Spalton J.A., Al Hikmani H., Robinson N.P., Almalki A., Gallacher E., Balme G.A., Robinson H.S. (2022). Modelling potential habitat suitability for critically endangered Arabian leopards (*Panthera pardus nimr*) across their historical range in Saudi Arabia. J. Nat. Conserv..

[4] Al-Jumaily M.M. (1998). Review of the mammals of the Republic of Yemen. Fauna Arab..

[5] Al-Johany A.M.H. (2007). Distribution and conservation of the Arabian Leopard Panthera pardus nimr in Saudi Arabia. J. Arid. Environ..

[6] Al Jumaily M., Mallon D.P., Nasher A.K., Thowabeh N. (2006). Status report on Arabian leopard in Yemen. Cat News.

[7] Al Ahmari A., Neyaz F., Shuraim F., Al Ghamdi A.R., Al Boug A., Alhlafi M., Al Jbour S., Angelici F.M., Alaamri S., Al Masabi K. (2024). Diversity and Conservation of Carnivores in Saudi Arabia. Diversity.

[8] Islam M., Boug A., Shehri A., Jackson R. (2017). National Strategy and Action Plan for the conservation of the Arabian Leopard in the Kingdom of Saudi Arabia. CATnews.

[9] Zafar-ul Islam M., Boug A., Judas J., As-Shehri A. (2018). Conservation challenges for the Arabian Leopard (Panthera pardus nimr) in the Western Highlands of Arabia. Biodiversity.

[10] Frankham R. (2005). Genetics and extinction. Biol. Conserv..

[11] Islam M., Boug A., As-Shehri A., Al Jaid M. (2015). Poisoning of endangered Arabian leopard in Saudi Arabia and its conservation efforts. Cat News.

[12] Mochales-Riaño G., Fontsere C., de Manuel M., Talavera A., Burriel-Carranza B., Tejero-Cicuéndez H., AlGethami R.H.M., Shobrak M., Marques-Bonet T., Carranza S. (2023). Genomics reveals introgression and purging of deleterious mutations in the Arabian leopard (*Panthera pardus nimr*). Iscience.

[13] Buddhirongawatr R., Tungsudjai S., Chaichoune K., Sangloung C., Tantawiwattananon N., Phonaknguen R., Sukthana Y. (2006). Detection of Toxolasma gondii in captive wild felids. Southeast Asian J. Trop. Med. Public Health.

[14] Stewart P., Campbell L., Skogtvedt S., Griffin K.A., Arnemo J.M., Tryland M., Girling S., Miller M.W., Tranulis M.A., Goldmann W. (2012). Genetic predictions of prion disease susceptibility in carnivore species based on variability of the prion gene coding region. PLoS ONE.

[15] Rampacci E., Diaferia M., Lucentini L., Brustenga L., Capasso M., Girardi S., Gizzi I., Primavilla S., Veronesi F., Passamonti F. (2024). Detection of zoonotic enteropathogens in captive large felids in Italy. Zoonoses Public Health.

[16] Avise J.C. (2000). Phylogeography: The History and Formation of Species.

[17] Boore J.L. (1999). Animal mitochondrial genomes. Nucleic Acids Res..

[18] Figueiró H.V., Li G., Trindade F.J., Assis J., Pais F., Fernandes G., Santos S.H.D., Hughes G.M., Komissarov A., Antunes A. (2017). Genome-wide signatures of complex introgression and adaptive evolution in the big cats. Sci. Adv..

[19] Uphyrkina O., Johnson W.E., Quigley H., Miquelle D., Marker L., Bush M., O’Brien S.J. (2001). Phylogenetics, genome diversity and origin of modern leopard, Panthera pardus. Mol. Ecol..

[20] Sooriyabandara M.G.C., Bandaranayake A.U., Hathurusinghe H., Jayasundara S., Marasinghe M., Prasad G.A.T., Abeywardana V.P.M.K., Pinidiya M.A., Nilanthi R.M.R., Bandaranayake P.C.G. (2023). A unique single nucleotide polymorphism in Agouti Signalling Protein (ASIP) gene changes coat colour of Sri Lankan leopard (*Panthera pardus kotiya*) to dark black. PLoS ONE.

[21] Ruiz-García M., Pinedo-Castro M., Shostell J.M. (2018). Mitogenomics of the jaguarundi (Puma yagouaroundi, Felidae, Carnivora): Disagreement between morphological subspecies and molecular data. Mamm. Biol..

[22] Uliano-Silva M., Ferreira J.G.R., Krasheninnikova K., Formenti G., Abueg L., Torrance J., Myers E.W., Durbin R., Blaxter M. (2023). MitoHiFi: A Python pipeline for mitochondrial genome assembly from PacBio high fidelity reads. BMC Bioinform..

[23] Bernt M., Donath A., Jühling F., Externbrink F., Florentz C., Fritzsch G., Pütz J., Middendorf M., Stadler P.F. (2013). MITOS: Improved de novo metazoan mitochondrial genome annotation. Mol. Phylogenetics Evol..

[24] Greiner S., Lehwark P., Bock R. (2019). OrganellarGenomeDRAW (OGDRAW) version 1.3.1: Expanded toolkit for the graphical visualization of organellar genomes. Nucleic Acids Res..

[25] Letunic I., Bork P. (2021). Interactive Tree Of Life (iTOL) v5: An online tool for phylogenetic tree display and annotation. Nucleic Acids Res..

[26] Desper R., Gascuel O. (2002). Fast and accurate phylogeny reconstruction algorithms based on the minimum-evolution principle. J. Comput. Biol..

[27] Robinson J.T., Thorvaldsdóttir H., Winckler W., Guttman M., Lander E.S., Getz G., Mesirov J.P. (2011). Integrative genomics viewer. Nat. Biotechnol..

[28] Li H. (2018). Minimap2: Pairwise alignment for nucleotide sequences. Bioinformatics.

[29] Li H., Handsaker B., Wysoker A., Fennell T., Ruan J., Homer N., Marth G., Abecasis G., Durbin R., 1000 Genome Project Data Processing Subgroup (2009). The Sequence Alignment/Map format and SAMtools. Bioinformatics.

[30] Tensen L., Camacho G. (2024). Dark Mystery Solved: A Captive Black Leopard from South Africa is of Asian Descent. Afr. J. Wildl. Res..

[31] Bertola L., Jongbloed H., Van Der Gaag K., De Knijff P., Yamaguchi N., Hooghiemstra H., Bauer H., Henschel P., White P., Driscoll C. (2016). Phylogeographic patterns in Africa and high resolution delineation of genetic clades in the lion (Panthera leo). Sci. Rep..

[32] Ren Z., Niu X., Lv T., Wang Y., Caraballo-Ortiz M.A., Su X. (2019). The complete mitochondrial genome of Panthera pardus (Felidae: Pantheriinae), a first-class national-protected wild animal from China. Conserv. Genet. Resour..

[33] Zhang M., Wang C.H., Zheng Y.X., Jiangzuo Q.G., Hou Y.M., Cao P., Dai Q.Y., Yang R.W., Liu F., Feng X.T. (2024). Ancient DNA unravels species identification from Laosicheng site, Hunan Province, China, and provides insights into maternal genetic history of East Asian leopards. Zool. Res..

[34] Dou H., Feng L., Xiao W., Wang T. (2016). The complete mitochondrial genome of the North Chinese Leopard (*Panthera pardus japonensis*). Mitochondrial DNA Part A.

[35] Kim J.A., Jeon H.S., Jeon J.H., Kim S., An J. (2019). Complete mitochondrial genome of the Amur leopard (*Panthera pardus orientalis* Schlegel, 1857). Mitochondrial DNA Part B.

[36] Pardinilla L.M., Muzaffar S., Giraldez A., Budd J.A., Al Aiyan A., Qablan M.A. (2024). Wild feline pathogens in the Arabian Peninsula: A review. J. Nat. Conserv..

[37] Spalton J.A., Al Hikmani H.M., Jahdhami M.H., Ibrahim A.A., Bait Said A., Willis D. (2006). Status report for the Arabian leopard in the Sultanate of Oman. Cat News.

[38] Judas J., Paillat P., Khoja A., Boug A. (2006). Status of the Arabian Leopard in Saudi Arabia. Cat News.

[39] Dunford C.E., Faure J.P.B., Ross M.D., Spalton J.A., Drouilly M., Pryce-Fitchen K.J., De Bruin R., Botha A.E., Alshehri A., Le Roex N. (2024). Searching for spots: A comprehensive survey for the Arabian leopard *Panthera pardus nimr* in Saudi Arabia. Oryx.

[40] Islam M.Z., Smith M., al Boug A. (2024). The decline of the Arabian Leopard *Panthera pardus nimr* in Saudi Arabia: A values-based plan for future management. Biodivers. Conserv..

[41] Allendorf F.W., Luikart G., Aitken S.N. (2012). Conservation and the Genetics of Populations.

[42] Tensen L., Emami-Khoyi A., Khan A., Camacho G., Swanepoel L., Fischer K. (2024). Mitogenomic Characterization of South African Leopards and the Effect of Past Climatic Events. J. Zool. Syst. Evol. Res..

[43] Wei L., Wu X., Jiang Z. (2009). The complete mitochondrial genome structure of snow leopard *Panthera uncia*. Mol. Biol. Rep..

[44] Wei L., Wu X., Zhu L., Jiang Z. (2011). Mitogenomic analysis of the genus *Panthera*. Sci. China Life Sci..

[45] Wilting A., Patel R., Pfestorf H., Kern C., Sultan K., Ario A., Peñaloza F., Kramer-Schadt S., Radchuk V., Foerster D. (2016). Evolutionary history and conservation significance of the Javan leopard Panthera pardus melas. J. Zool..

[46] Paijmans J.L., Barlow A., Förster D.W., Henneberger K., Meyer M., Nickel B., Nagel D., Worsøe Havmøller R., Baryshnikov G.F., Joger U. (2018). Historical biogeography of the leopard (*Panthera pardus*) and its extinct Eurasian populations. BMC Evol. Biol..

[47] Hyun J.Y., Cho J.H., Pandey P., Min M.S., Kim K.S., Lee H. (2020). Phylogenetic study of extirpated Korean leopard using mitochondrial DNA from an old skin specimen in South Korea. PeerJ.

[48] Atzeni L., Ilany A., Geffen E., Cushman S.A., Kaszta Ż., Macdonald D.W. (2024). Reviving the Arabian leopard: Harnessing historical data to map habitat and pave the way for reintroduction. Biol. Conserv..

[49] Alqahtani F., Măndoiu I. (2019). SMART2: Multi-library statistical mitogenome assembly with repeats. Proceedings of the International Conference on Computational Advances in Bio and Medical Sciences.

[50] Dierckxsens N., Mardulyn P., Smits G. (2017). NOVOPlasty: De novo assembly of organelle genomes from whole genome data. Nucleic Acids Res..

